# CPT1a gene expression reverses the inflammatory and anti-phagocytic effect of 7-ketocholesterol in RAW264.7 macrophages

**DOI:** 10.1186/s12944-019-1156-7

**Published:** 2019-12-10

**Authors:** Priscila Calle, Angeles Muñoz, Anna Sola, Georgina Hotter

**Affiliations:** 1 Department of Experimental Pathology, Institut d’Investigacions Biomèdiques de Barcelona-Consejo Superior de Investigaciones Científicas-Institut d’Investigacions Biomèdiques August Pi i Sunyer (IIBB-CSIC-IDIBAPS), Rosselló 161, 7th floor, 08036 Barcelona, Spain; 2 Department of Experimental Nephrology, Institut d’Investigació Biomèdica de Bellvitge (IDIBELL), Hospitalet de Llobregat, Barcelona, Spain; 3 Centro de Investigación Biomédica en Red en Bioingeniería, Biomateriales y Nanomedicina. (CIBER-BBN), Barcelona, Spain

**Keywords:** Macrophage, 7-ketocholesterol, CPT1a, Phagocytosis, Inflammation

## Abstract

**Background:**

Macrophage are specialized cells that contributes to the removal of detrimental contents via phagocytosis. Lipid accumulation in macrophages, whether from phagocytosis of dying cells or from circulating oxidized low-density lipoproteins, alters macrophage biology and functionality. It is known that carnitine palmitoyl transferase 1-a (CPT1a) gene encodes an enzyme involved in fatty acid oxidation and, therefore, lipid content. However, the potential of CPT1a to activate macrophage phagocytic function have not been elucidated.

**Methods:**

Using a murine macrophage cell line, RAW264.7, we determine if intracellular accumulation of 7-ketocholesterol (7-KC) modulates macrophage phagocytic function through CPT1a gene expression. In addition, the effects of CPT1a genetic modification on macrophage phenotype and phagocytosis has been studied.

**Results:**

Our results revealed that CPT1a gene expression decreased by the accumulation of 7-KC at the higher dose of 7-KC. This was concomitant with an impair ability to phagocytize bioparticles and an inflammatory phenotype. GW3965 treatment, which have shown to facilitate the efflux of cholesterol, eliminated the intracellular lipid droplets of 7-KC-laden macrophages, increased the gene expression of CPT1a, diminished the gene expression of the inflammatory marker iNOS and restored macrophage phagocytosis. Furthermore, CPT1a Knockdown per se was detrimental for macrophage phagocytosis whereas transcriptional activation of CPT1a heightened the uptake of bioparticles.

**Conclusions:**

Altogether, our findings indicate that downregulation of CPT1a by lipid content modulates macrophage phagocytosis and inflammatory phenotype.

## Background

Macrophages are professional ﻿phagocytes aimed to remove pathogens, small particles as well as damaged or dead cells [1]. If macrophage phagocytosis is compromised, neighboring cells will engulf apoptotic cells at a much slower rate, prolonging exposure to cytotoxic contents, thereby perpetuating an inflammatory state [2]. As macrophages take up lipoproteins acquired due to the phagocytosis of dying cells, they have evolved mechanisms for eliminating cholesterol from the cell. If excess cholesterol is not eliminated from macrophages, the transformation into foam cells could occur. Such foam cells are a hallmark of the atherosclerotic lesion contributing to the development and rupture of atherosclerotic plaques [3]. In addition, macrophages also internalize low-density lipoproteins (LDL), very low-density lipoproteins (VLDL) and oxidized low-density lipoprotein (oxLDL) via phagocytosis, macropinocytosis or scavenger receptors such as CD36 [4]. Once internalized, lipids are degraded in the lysosome by lysosomal acid lipase [5] into free cholesterol and fatty acids [6]. In the cytosol, fatty acids are activated to acyl-coenzyme A (acyl-CoA) by acyl-CoA synthetases, either for lipid biosynthesis or for mitochondrial fatty acid β-oxidation (FAO) [7]. As the mitochondrial membrane is impermeable to acyl-CoAs, the carnitine shuttle manages their transport into the mitochondria. First, on the outer mitochondrial membrane, the enzyme carnitine palmitoyltransferase-1 (CPT1) converts acyl-CoA to acylcarnitine. Next, this complex is translocated across the inner mitochondrial membrane to the mitochondrial matrix by the carnitine-acylcarnitine translocase and finally reconverted back to acyl-CoA by the enzyme CPT2 of the peripheral inner mitochondrial membrane [8]. It has been shown that the expression of a permanently active mutant of CPT1a enhanced fatty acid oxidation in macrophages and reduced pro-inflammatory cytokines [9], suggesting that inducing FAO in foam cells could be of therapeutic potential. It seems that the expression of CPT1a has an influence on inflammation and on the elimination of intracellular lipids. However, the relationship between CPT1a, foam cells formation and macrophage phagocytic function remains unclear.

Herein 7-KC was used to induce high lipid load macrophages, since is among the most abundant oxysterol to be found in oxLDL [10] and atherosclerotic plaques [11]. We converted RAW264.7 into foam cells by 7-KC accumulation and examined the alterations in CPT1a gene expression, modifications on macrophage inflammatory phenotype and the uptake of pHrodo bioparticles. In addition, the direct effects of CPT1a expression on macrophage phenotype and phagocytosis were studied throughout genetic strategies of knockdown and transcriptional activation of CPT1a.

Our results reveal that macrophage phagocytosis and inflammatory phenotype are dependent on intracellular lipid accumulation and CPT1a expression, and that the direct downregulation of CPT1a by high lipid content in macrophages is a key modulator of this process.

## Methods

### Materials

Dulbecco’s Modified Eagle Medium/Nutrient Mixture F-12 with GlutaMAX (DMEM/F12 + GlutaMAX), Heat Inactivated Fetal Bovine Serum (FBS) and Penicillin/Streptomycin were obtained from Gibco (Madrid, Spain). Adenovirus vector with a short hairpin RNA target to CPT1a and the control non-targeted were generated by Galapagos NV (Mechelen, Belgium). Plasmid Transfection Medium, UltraCruz Transfection Reagent, Control CRISPR Activation Plasmid and CPT1 CRISPR Activation Plasmid were obtained from Santa Cruz Biotechnology, Inc. (Heidelberg, Germany). Live cell image solution and pHrodo Green *E. coli* BioParticles conjugate were purchased from Molecular Probes (Madrid, Spain). 7-Ketocholesterol (5-Cholesten-3β-ol-7-one) and GW3965 hydrochloride were obtained from Sigma-Aldrich (Madrid, Spain). C75 (4-Methylene-2-octyl-5-oxotetrahydrofuran-3-carboxylic acid) was purchased from Santa Cruz Biotechnology, Inc. (Heidelberg, Germany). 7-ketocholesterol stock solution was prepared by dissolving the chemical compound in 100% ethanol at concentration of 5 mg/ml. C75 and GW3965 stock solutions were prepared in dimethyl sulfoxide (DMSO) at a concentration of 10 mg/ml and 32,34 mM respectively. Designed primers were bought from Life Technologies (Madrid, Spain).

### Cell culture and treatments

The murine macrophage cell line RAW264.7 (obtained from the European Collection of Authenticated Cell Cultures) was cultured in DMEM/F12 + GlutaMAX supplemented with 10% FBS and 1% antibiotics (100 Units/ml penicillin and 100 μg/ml streptomycin). Cells were maintained in a humidified incubator at 37 °C under 5% CO_2_ and passaged twice a week reaching 80% confluence by cell scraping. Cells from passage 10–15 were culture in 12-well plate and allow to grow for 24 h until reach the desired confluence for each procedure.

RAW264.7 grown to 60–80% were incubated with 7-KC at 5 μg/ml and 10 μg/ml for 24 h in DMEM/F12 + GlutaMAX supplemented with 10% FBS and 1% antibiotics. GW3965 treatment was at 3 μM for 16 h following 7-KC incubation and C75 pre-treatment was administrated at 10μg/ml for 2 h before 7-KC incubation. Control cells were incubated with the corresponding vehicles.

### shRNA adenoviral transduction

RAW264.7 macrophages grown to 80% confluence were transduced with adenovirus vector carrying a short hairpin targeting CPT1a with a multiplicity of infection of 150 in antibiotic free DMEM/F12 + GlutaMAX supplemented with 2% FBS and 1 μl of poly-l-lysine for 24 h. A non-targeted short hairpin RNA was used as negative control. Cells were used 24 h post transduction.

### CRISPR activation plasmid transfection

RAW264.7 macrophages were grown to 60–80% confluence in antibiotic free DMEM/F12 + GlutaMAX supplemented with 10% FBS and transfected with CPT1a CRISPR activation plasmid (sc-419786-act) and control CRISPR activation plasmid (sc-437,275) according to the manufacturer’s protocol. In brief, 0.5 μg of plasmid DNA and 5 μl of UltraCruz transfection reagent (sc-395739) were used for each transfection. After 24 h, the relevant assays were performed.

### Oil red O staining (ORO)

To prepare the ORO working solution, 3 parts of 0.3% ORO was mixed with 2 parts of distillated water, allowed to settle for 10 min and filtered before use. Cells were fixed with 10% formalin for 10 min, washed with PBS, rinsed with 60% isopropanol for 5 min and stained with ORO working solution for 5 min. Nuclei were counterstained with hematoxylin. Images were acquired in a Zeiss Axiophot microscope and analyzed using ImageJ2. Quantitative Oil Red O staining was performed by eluting the dye with isopropanol. Briefly, the dye was eluted from the stained cells using 500 μl of 100% isopropanol for 20 min in an orbital shaker. Then, two aliquots of 200 μl were transferred to a 96-well plate and the absorbance was read at 510 nm in a Multiskan Sky Microplate Spectrophotometer (Thermo Fisher Scientific, USA).

### Phagocytosis assay

One vial of pHrodo Green *E. coli* BioParticles conjugate (P35366) was suspended in 2 ml of LCIS (Live Cell Imaging Solution, A14291DJ) at 1 mg/ml, thoroughly vortexed and sonicated following manufacturer’s instructions. Bioparticles were tittered to a final concentration of 55 μg/ml per well for the assay. After each experiment, RAW264.7 cells were incubated with pHrodo Green *E. coli* BioParticles diluted in LCIS at 55 μg/ml for 90 min at 37 °C. Cell imaging was performed on Leica CTR 4000 microscope and fluorescence intensity was measured using a Spectramax Gemini XS spectrofluorometer plate reader (Molecular Devices, Sunnyvale, CA) at excitation 485 nm and emission 530 nm. As indicated by the manufactures, net phagocytosis was calculated by subtracting the average fluorescence intensity of the no-cell negative control wells from all sample wells. Net phagocytosis data are presented as the change level relative to the observed in the control untreated cells.

### Quantitative real-time polymerase chain reaction (qPCR)

Total RNA was isolated using the RNeasy Mini kit (Qiagen). A total among of 1μg RNA was reverse transcribed into cDNA using the iScript cDNA synthesis Kit (Bio-Rad). qPCR assay was performed on a Bio-Rad CFX96 Touch Real Time PCR detection system with SooAdvanced Universal SYBR Green Supermix (Bio-Rad). Sequences of specific primers are listed in Table 1. The housekeeping gene GAPDH was used as internal control. Reactions were carried in triplicate. qPCR data were analyzed using the relative mRNA expression method calculated by the 2^-ΔΔCT^ equation, where ΔCT = Ct _(target gene) –_ Ct _(gapdh)_ and ΔΔCT = ΔCt _(treated) –_ ΔCt _(untreated)._ All data are presented as mRNA expression levels relative to untreated control values.

**Table 1:** Primers sequences used for qPCR

Gene	Forward primer	Reverse primer
*GAPDH*	TGAAGCAGGCATCTGAGGG	CGAAGGTGGAAGAGTGGGAG
*CPT1a*	TTTGAATCGGCTCCTAATGG	CCCAAGTATCCACAGGGTCA
*CD36*	CAGCTCATACATTGCTGTTTATGCATG	GGTACAATCACAGTGTTTTCTACGTGG
*iNOS*	AGGGAATCTTGGAGCGAGTT	GCAGCCTCTTGTCTTTGACC
*NLRP3*	Assay ID: qMmuCID0010647. PrimePCR™ SYBR® Green Assay (Bio-Rad).	

*GAPDH* glyceraldehyde 3-phosphate dehydrogenase, *CPT1a* carnitine palmitoyl transferase 1-a, *CD36* cluster of differentiation CD36, *iNOS* inducible nitric oxide synthase, *NLRP3* NOD-like receptor pyrin domain-containing-3

### Statistical analysis

All data were reported as mean ± SE of at least three independent experiments, each experiment with an *n* = 3. Unpaired t-test was used to compare means among two groups. Differences in values were considered to be statistically significant if *P* < 0.05. Statistical analyses were performed with GraphPad Prism 8.0 software.

## Results

### Intracellular lipid content modifies macrophage phenotype, CPT1a gene expression and phagocytosis

To understand the effect of intracellular lipid content on macrophage phenotype, CPT1a expression and phagocytosis, RAW264.7 murine macrophages were incubated with 7-KC at 5 and 10 μg/ml for 24 h as previously reported [12] to obtain foam cells. Additionally, to test the effect of lipid reduction in 7-KC overloaded macrophages, lipid efflux was promoted by treatment with 3 μM of GW3965 (GW) for 16 h following 7-KC administration. Control cells were incubated with ethanol for 24 h following DMSO for 16 h. We evaluated the intracellular lipid droplets stained by oil red-o, mRNA expression levels by qPCR and the phagocytosis by pHrodo Green *E. coli* bioparticles.

**Fig. 1:**
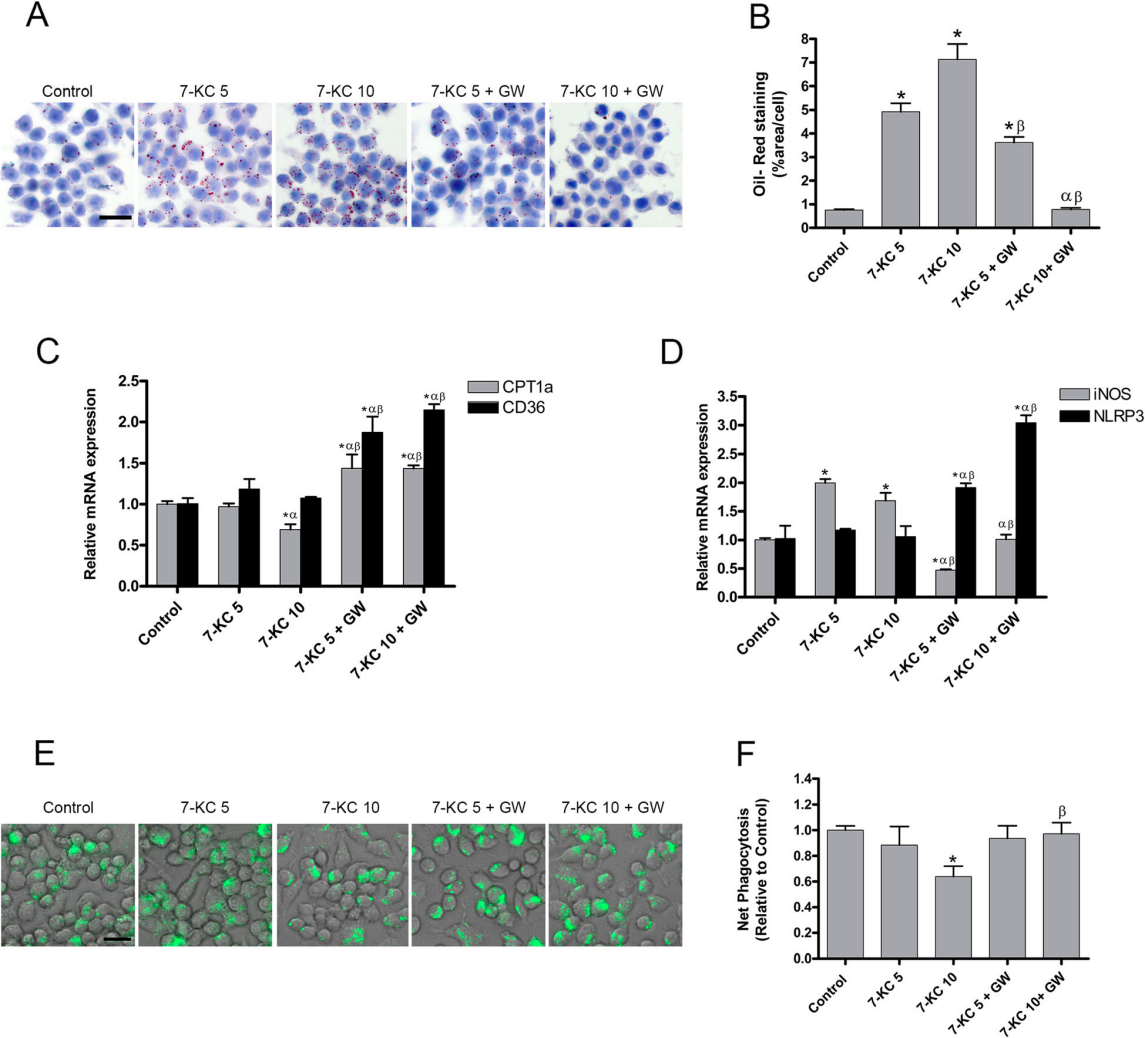
Dose-dependent effects of 7-Ketocholesterol on macrophage lipid content, phenotype and phagocytosis. RAW264.7 were incubated with 7-ketocholesterol at 5 μg/ml (7-KC 5) and 10 μg/ml (7-KC 10) for 24 h, following treatment with 3 μM of GW3965 for 16 h (7-KC 5 + GW) and (7-KC 10 + GW). As control, cells were incubated with ethanol for 24 h following DMSO for 16 h (Control). **a** Representative Oil Red O staining images, scale bar 20 μm. **b** Quantification of oil red by determining absorbance at 510 nm after elution with isopropanol. **c**, **d** qPCR analysis of mRNA expression levels represented relative to control of the experiments (**c**) CPT1a and CD36 (**d**) iNOS and NLRP3. **e** Net Phagocytosis of 55 μg/ml pHrodo Green E. ecoli bioparticles conjugate for 90 min measured by spectrofluorometer at excitation 485 nm and emission 530 nm. **f** Representative images of pHrodo uptake capture by fluorescence microscopy, scale bar 20 μm. Data are represented as means ±SEM of 3 independent experiments with *n* = 3 each. * ≤ 0.05 versus Control; α ≤ 0.05 versus 7-KC 5; β ≤ 0.05 versus 7-KC 10

As shown in Fig. 1a, b, the incubation with 7-KC in macrophages, leaded to an accumulation of intracellular lipid droplets that was more pronounced when exposed to 7-KC at 10 μg/ml whereas GW treatment diminished this accumulation of lipids, as represented by a decrease in oil red o-stained lipids.

As shown in Fig. 1c, CPT1a mRNA expression remained unmodified when cells were exposed to 7-KC at 5 μg/ml (7-KC 5) but was significantly down-regulated at the dose of 10 μg/ml (7-KC 10). In contrast, GW treatment significantly up-regulated CPT1a expression in both 7-KC concentrations. These results suggested that CPT1a expression increases when the intracellular lipid accumulation diminish. Similarly, CD36 mRNA expression was up-regulated after GW treatment, also regardless of the initial dose of 7-KC. Thus, a decrease of lipid content not only increases the expression of CPT1a but also of the scavenger receptor CD36. Moreover, 7-KC-induced intracellular lipid accumulation shifted macrophage towards a pro-inflammatory phenotype by enhancing iNOS mRNA expression and treatment with GW reversed this effect (Fig. 1d). Hence, a decline in inflammatory phenotype is concomitant with a reduction in lipid content and CPT1a gene expression is involved. NLRP3 (Fig. 1d) also heightened as a consequence of GW treatment, suggesting that CPT1a expression and NLRP3 are related.

In order to evaluate macrophage phagocytic ability, RAW264.7 were incubated with 55 μg/ml of pHrodo Green *E. coli* bioparticles for 90 min at 37 °C. As shown in Fig. 1e, f, only macrophages exposed at 7-KC10 significantly lessened the uptake of pHrodo bioparticles. Thus, 7-KC impairs phagocytosis at the dose in which CPT1a is decreased. GW treatment had no effect on phagocytosis in cells exposed to 7-KC 5 (7KC5 + GW), however it did increase the uptake of pHrodo bioparticles to basal conditions in cells previously incubated with 7-KC10 (7KC10 + GW). Taken together, these results reveal that phagocytosis is compromised by intracellular lipid content only if CPT1a expression is decreased.

Thereafter, we pharmacologically target CPT1a with the compound C75 which is a CPT1 activity inhibitor (Bentebibel et al. 2006). The above findings presented that 7-KC at the concentration of 10 μg/ml resulted in a decrease of CPT1 expression; therefore, we incubated RAW264.7 with 7-KC at 10 μg/ml for 24 h in the absence or presence of C75 pre-treatment at 10 μg/ml for 2 h. Control cells were incubated with DMSO for 2 h following ethanol for 24 h.

**Fig. 2:**
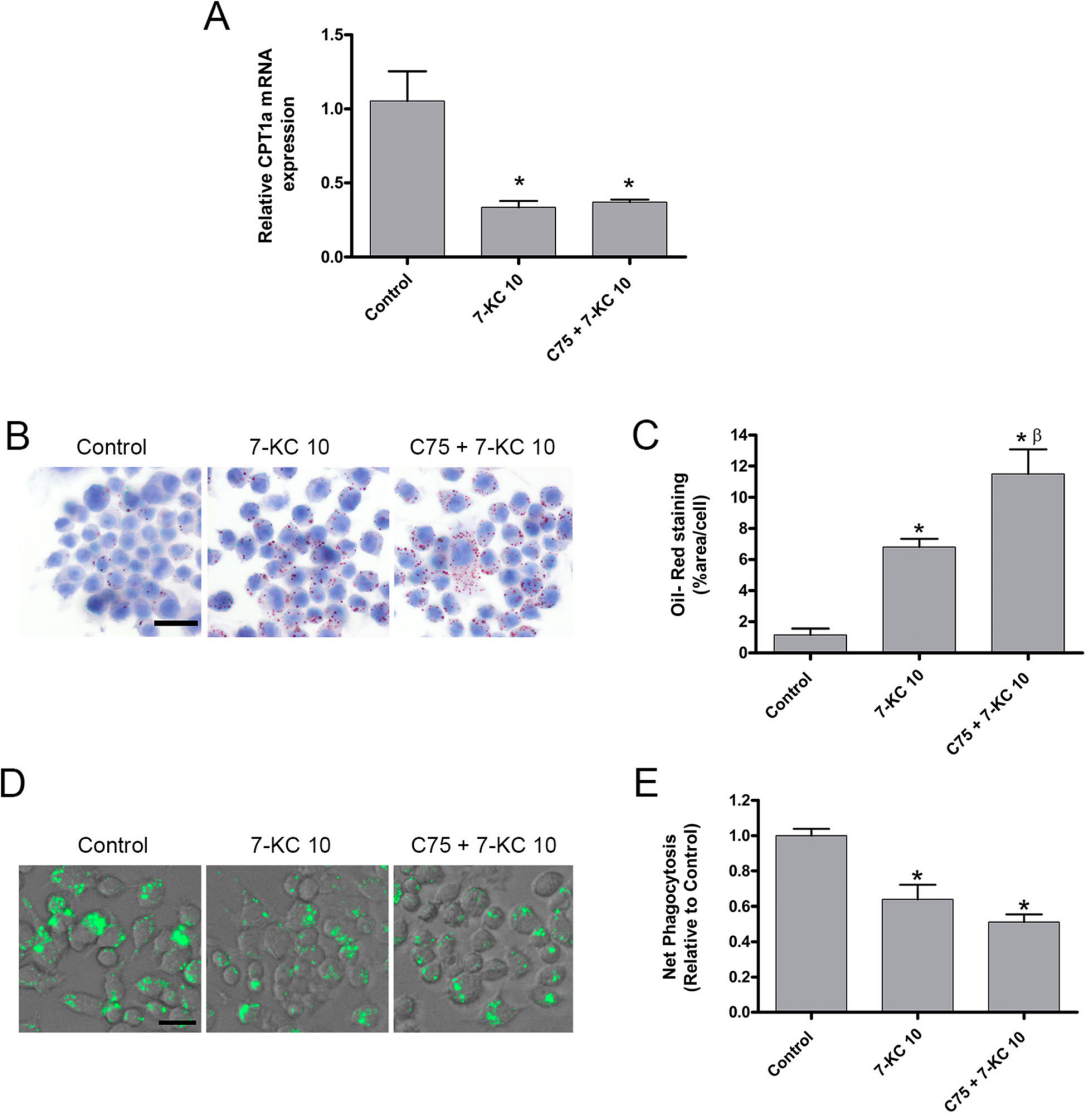
7-Ketocholesterol impairs phagocytosis through CPT1a gene expression. RAW264.7 were incubated with 7-ketocholesterol at 10 μg/ml (7-KC10) for 24 h in the absence or presence of C75 pre-treatment at 10 μg/ml for 2 h (C75 + 7-KC10). (Control) cells were incubated with DMSO for 2 h before ethanol incubation for 24 h . (C75) cells were incubated with C75 at 10 μg/ml for 2 h before ethanol incubation for 24 h. **a** CPT1a mRNA expression determined by qPCR, presented as relative to controls **b** Representative Oil Red O staining images, scale bar 20 μm. **c** Quantification of oil red by determining absorbance at 510 nm after elution with isopropanol. **d** Net phagocytosis of 55 μg/ml pHrodo Green *E. coli* bioparticles conjugate for 90 min measured by spectrofluorometer at excitation 485 nm and emission 530 nm. **e** Representative images of pHrodo uptake capture by fluorescence microscopy, scale bar 20 μm. Data are represented as means ±SEM of 4 independent experiments. * ≤ 0.05 versus Control; β ≤ 0.05 versus 7-KC 10.

Figure 2a, show a decreased expression of CPT1a by 7-KC10 treatment, which in this case is more pronounced than that observed in Fig. 1c, this is because the experimental conditions of the control group are different in the two experiments and this influences the expression of CPT1a, therefore the same CPT1a expression value induced by 7-KC10 gives rise to different expressions relative to controls in the two experiments.

As shown in Fig. 2a, C75 pre-treatment maintained the down-regulated expression of CPT1a induced by 7-KC.

When the lipid content was evaluated by oil red-o staining, C75 pre-treatment significantly heightened lipid droplets respect 7-KC exposure alone (Fig. 2c). Indicating that a decrease in CPT1a activity due to C75 promotes a higher accumulation of neutral lipids in the cytosol, but was unable to modify CPT1a expression. In addition, C75 pre-treatment did not change by any means the impaired phagocytosis produced by an increased intracellular lipid content due to 7-KC exposure (Fig. 2d, e). In addition, C75 administration to control cells did not modify any of the measured parameters. Taken together, these observations suggest that increases in lipid droplets accumulation (as occurs in C75 + 7KC-10 with respect to 7KC-10) not always lead to a decrease phagocytosis and that there are more factors that modulate it.

### CPT1a knockdown impairs phagocytosis and stimulates inflammation

In order to evaluate the role of CPT1a on macrophage phenotype and phagocytosis, RAW264.7 cell line was transduced with either adenovirus encoding a short hairpin CPT1a-target (Adsh CPT1a) or a non-target short hairpin (AdshCtrl) for 24 h and transduction efficacy was confirmed by qPCR. As control, un-transduced cells were used.

**Fig. 3:**
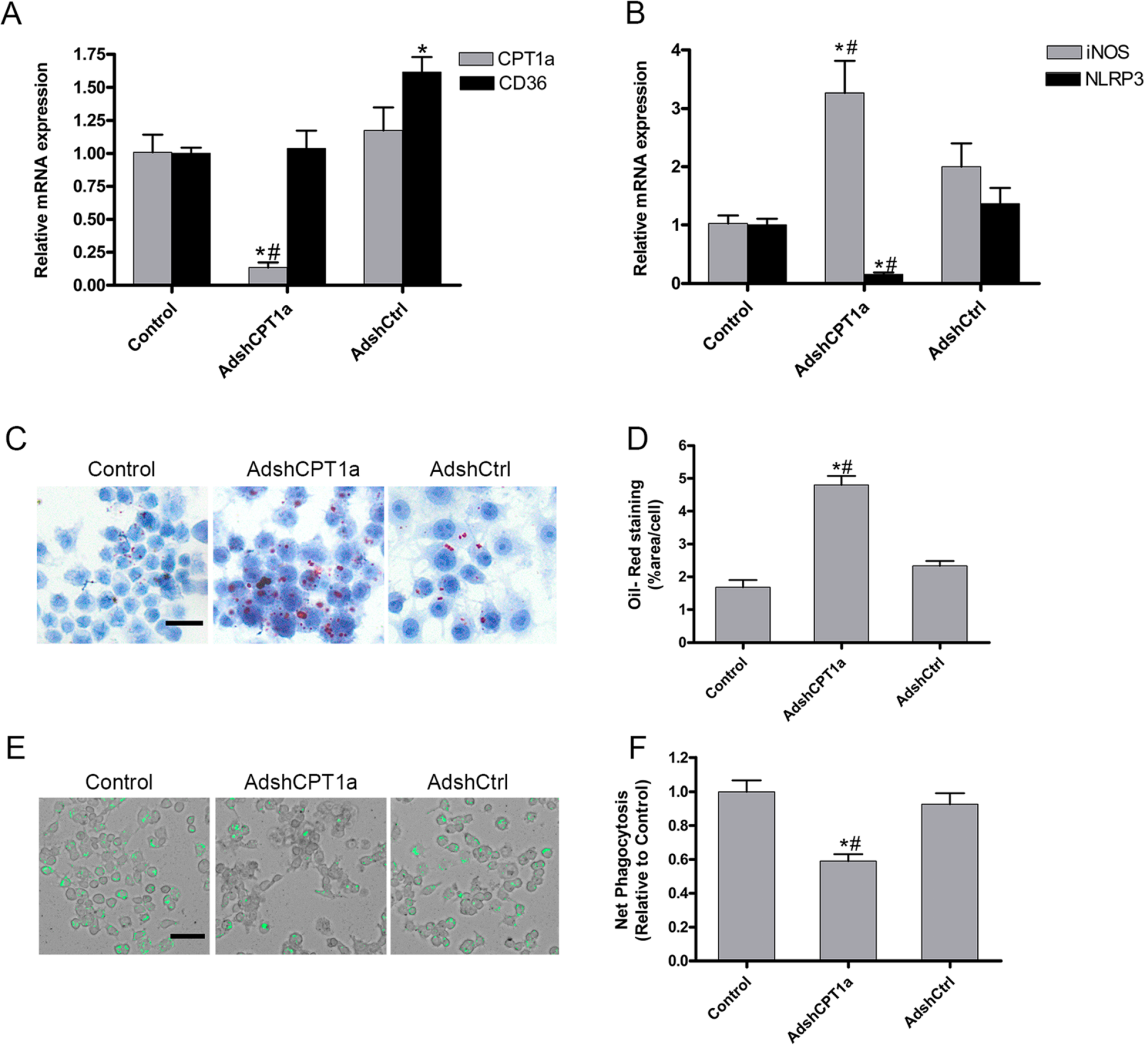
CPT1a Knockdown is involved in macrophage phenotype and phagocytosis. RAW264.7 were transduced with adenovirus encoding a short hairpin RNA targeting CPT1a (AdshCPT1a) and a non-target short hairpin RNA (AdshCtrl) for 24 h, as control un-transfected macrophages were used (Control). **a, b** mRNA expression determined by qPCR of (**a**) CPT1a and CD36 (**b**) iNOS and NLRP3. **c** Representative Oil Red O staining images, scale bar 20 μm. **d** Quantification of oil red o staining images. **e** Net Phagocytosis of 55 μg/ml pHrodo Green E. ecoli bioparticles conjugate for 90 min measured by spectrofluorometer at excitation 485 nm and emission 530 nm. **f** Representative images of pHrodo uptake capture by fluorescence microscopy, scale bar 50 μm. Data are represented as means ±SEM of 4–5 independent experiments with *n* = 3 each. * ≤ 0.05 versus Control; # ≤ 0.05 versus AdshCtrl

As shown in Fig. 3a, CPT1a Knockdown on macrophages did not modify CD36 expression respect to un-transduced cells but it did promote an up-regulation of the pro-inflammatory marker iNOS (Fig. 3b), suggesting that CPT1a expression may be needed to avoid a pro-inflammatory phenotype. Interestingly, CPT1a silencing provoked a decrease on NLRP3 expression (Fig. 3b).

To evaluate macrophages intracellular lipid content, post-transduced cells were stained with Oil Red O. As shown in Fig. 3c, d, CPT1a knockdown macrophages exhibited increased intracellular lipid droplets compared to AdshCtrl and un-transduced cells (Control). In addition, the phagocytic function demonstrated that CPT1a gene silencing leaded to a significant decrease in phagocytosis (Fig. 3e, f).

Overall, results indicate that CPT1a knockdown promotes an increase in lipid accumulation and in the pro-inflammatory marker iNOS associated to an impaired phagocytosis and down-regulation of NLRP3.

### CPT1a overexpression decreases lipid content and stimulates phagocytosis

Furthermore, to elucidated the effect of CPT1a expression on lipid accumulation and phagocytosis ability we transfected RAW264.7 cells with either CPT1a Crispr activation plasmid (CPT1a Crispr) or Control Crispr activation plasmid (Control-Crispr) for 24 h.

**Fig. 4:**
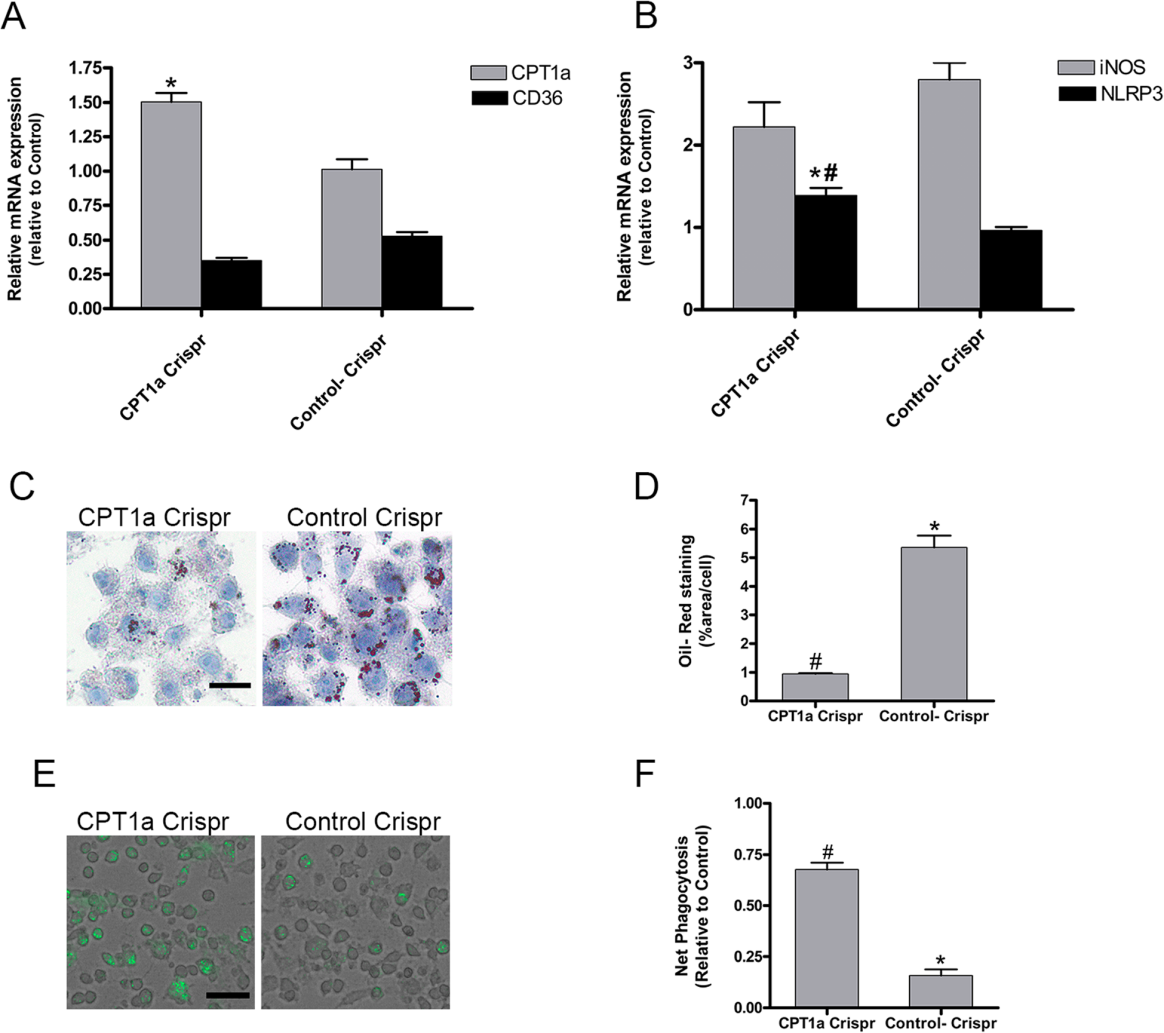
CPT1a overexpression decreases lipid content and stimulates phagocytosis. RAW264.7 were transfected with either CPT1a CRISPR activation plasmid (CPT1a Crispr) or Control CRISPR activation plasmid (Control Crispr) for 24 h. **a, b** mRNA expression measure by qPCR of (**a**) CPT1a and CD36 (**b**) iNOS and NLRP3. **c** Representative Oil Red O staining images, scale bar 20 μm. **d** Quantification of oil red o staining images. **e** Net Phagocytosis of 55 μg/ml pHrodo Green E. ecoli bioparticles conjugate for 90 min measured by spectrofluorometer at excitation 485 nm and emission 530 nm. **f** Representative images of pHrodo uptake capture by fluorescence microscopy, scale bar 50 μm. All data are represented as means ±SEM of 3 independent experiments with *n* = 3 each. * ≤ 0.05 versus Control; # ≤ 0.05 versus Control- Crisp

As shown in Fig. 4a, qPCR analysis showed that macrophages transfected with CPT1a plasmid had an increase in CPT1a mRNA expression respect to Control Crispr cells. mRNA expressions of CD36 and iNOs showed no significantly difference between both transduced cells (Fig. 4a, b), while significant increases were observed in NLRP3 expression. As shown in Fig. 4b, the use of a control crisp activation plasmid induces an iNOS expression that is three times higher than the control value, indicating a non-specific effect of the plasmid, but the use of the control crisp activation plasmid affects neither NPLR3 expression nor CPT1a expression.

The imaging of neutral lipids by oil red displayed an accumulation of lipid droplets in the cytosol of Control Crispr whereas the lipid content in on macrophages expressing CPT1a was significantly lesser (Fig. 4c, d). The ability to uptake pHrodo bioparticles was enhanced in CPT1a Crisp regard to Control-Crispr (Fig. 4e, f).

Together these findings indicated that CPT1 expression is not only related to the accumulation of intracellular lipid droplets but is also involved in the macrophage phagocytic function.

## Discussion

Macrophages are well known for their plasticity, adapting their phenotype in response to internal and environmental cues. The most recognized nomenclature derives from in vitro stimulation and classifies macrophages into classically activated or pro-inflammatory (M1) and alternatively activated or anti-inflammatory (M2) macrophages. Although in vivo, this dichotomous M1/M2 classification system may not apply since the combination of stimulus are more complex, as demonstrated in human monocyte-derived macrophages by adding other conditions apart from the standard M1 or M2 polarization such as high-density lipoproteins (HDL), free fatty acids or a combination of stimuli associated with chronic inflammation [13]. Hence, a model were macrophages display a mixed phenotype rather than a M1 versus M2 usual phenotype, is most likely [14].

In our study, the accumulation of intracellular lipids induced by 7-KC stimulated the mRNA expression of the pro-inflammatory marker iNOS consistent with previous reports of 7-KC as an pro-inflammatory inductor [15]. Notably, only at the higher concentration of 7-KC, iNOS up-regulation is concomitant with CPT1a down-regulation whereas CD36 and NLRP3 mRNA expression remained unchanged.

The compound GW3965, a synthetic liver X receptor (LXR) ligand, activates the transcription of genes encoding ATP-binding cassette transporters A1 and G1 which have shown to facilitate the efflux of cholesterol [16] and 7-KC [17] in macrophages xs. Additionally, it has been shown that the synthetic ligand-activation of LXR by GW inhibits the induction of inflammatory genes in response to M1 stimuli on thioglycollate-elicited mouse peritoneal macrophages [18]. When we treated 7-KC laden macrophage with GW, as expected, the intracellular lipid content decreased and the inflammatory state reverted. Interestingly, CPT1a mRNA expression increased in conjunction with CD36 and NLRP3. Reports reveal that M2 macrophages has an increase expression of scavenger receptor CD36 [19]. So, there seems to be a correlation between the stimulation of CPT1a expression and a switch of M1 phenotype towards M2. Similarly, the uptake of pHrodo bioparticles by lipid-laden macrophages changed in a similar pattern of those observed in CPT1a expression, as phagocytosis impaired when CPT1a decreased, due to the excess in lipid content, and recovered when CPT1a increases, after lipid efflux by GW.

Cellular metabolism also contributes to macrophage activation, as it has been noticed that M1 rely on glycolysis whereas M2 seems to prefer fatty acid oxidation and glutamine metabolism as energy source [20–22]. It appears that the presence or absence of lipids has a great impact on macrophage biology, since macrophages have been implicated in the pathogenesis of diseases where lipid homeostasis is perturbed such as atherosclerosis, non-alcoholic fatty liver disease [3] and chronic kidney disease [23]. Lipids from LDL, VLDL and oxLDL taken up by macrophages are degraded through lysosomal lipolysis into free cholesterol and fatty acids and eventually exported to HDL or transported into the mitochondria, respectively. Fatty acids are transported to the mitochondria via carnitine shuttle system for energy production through FAO, whereby CPT1 is consider to be the rate-limiting enzyme in FAO [24]. It has been reported, that the expression of a malonyl-CoA insensitive form of CPT1A on RAW264.7 macrophages, not only enhanced FAO but also reduced the production of pro-inflammatory cytokines from palmitate-induction [9]. It seems interesting to determine whether FAO has a correlative or causal role in macrophage polarization.

In our study we use the compound C75, since it has been shown to be converted within the cell to its coenzyme-A derivative (C75-CoA), which directly inhibits CPT1 activity [25, 26]. When we pharmacologically targeted CPT1a with C75 before 7-KC exposure, the accumulation of lipid droplets significantly increased respect to 7KC-overload macrophages, as a response of the enzymatic activity inhibition of CPT1, although CPT1a expression and phagocytosis remained attenuated. These findings suggest that modifications in FAO may not affect CPT1a expression or phagocytosis.

Our results found, that the conversion of macrophages into foam cells provoked a deficient uptake of bioparticles when CPT1a expression was down-regulated. Moreover, the formation of foam cells was accompanied by an inflammatory phenotype and it is known that cholesterol crystals induce the activation of NLRP3 inflammasome [27]. Recently, it has been proved that intracellular lipid overload derived from excessive cholesterol-rich myelin, actives NLRP3 inflammasome [28]. By the contrary, in our study, the reduction of 7-KC-overload in the cytosol by GW-treatment potentiated the expression of the inflammasome related gene NLRP3.

The fact that other authors have observed a NOX4 dependent inactivation of CPT1a leading to a reduction in NLRP3 activation in macrophages [29] could help to explain our findings, since GW-treatment stimulate an increased CPT1a expression that could be responsible for the ﻿increased expression NLRP3. Moreover, CPT1a knockdown correlated with a decrease in NLRP3 expression, hence, a direct relationship could be suggested.

Furthermore, strategies on CPT1a genetic modification confirmed the above findings. In CPT1a knockdown macrophages, lipid droplets were found increased, together with up-regulated iNOS expression leading to an inflammatory state where phagocytosis was found to be impaired. In accordance, CPT1a overexpression by Crispr activation maintained a basal content of lipid droplets with an improved phagocytosis respect Control Crispr plasmid.

Although we did not find a shift towards an anti-inflammatory phenotype when CPT1a was overexpressed, since no significant differences were found in CD36 and iNOS; thus, indicating that macrophages phagocytosis is not a simple anti- or pro-inflammatory response, highlighting their heterogeneity.

## Conclusion

7-KC10 is able to downregulate CPT1a expression leading to an inflammatory phenotype and phagocytosis impairment. Whereas lipid content reduction stimulated CPT1a expression, reducing the inflammatory phenotype and improving phagocytosis.

Altogether, our findings reveal a causative link between CPT1a expression and macrophage phagocytic ability. Our results reveal that downregulation of CPT1a by lipid content modulates macrophage phagocytosis and inflammatory phenotype.

## Abbreviations

7-KC: 7-Ketocholesterol
CD36: cluster of differentiation 36
CPT1a: carnitine palmitoyl transferase 1-a
FAO: fatty acid β-oxidation
GW: GW3965
iNOS: inducible nitric oxide synthase
NLRP3: NOD-like receptor pyrin domain-containing-3

## References

[1] Schrijvers DM, De Meyer GRY, Herman AG, Martinet W (2007). Phagocytosis in atherosclerosis: molecular mechanisms and implications for plaque progression and stability. Cardiovasc Res.

[2] Krysko DV, D’Herde K, Vandenabeele P (2006). Clearance of apoptotic and necrotic cells and its immunological consequences. Apoptosis.

[3] Remmerie A, Scott CL (2018). Macrophages and lipid metabolism. Cell Immunol.

[4] Tabas I, Bornfeldt KE (2016). Macrophage phenotype and function in different stages of atherosclerosis. Circ Res.

[5] Huang SCC, Everts B, Ivanova Y, O’Sullivan D, Nascimento M, Smith AM (2014). Cell-intrinsic lysosomal lipolysis is essential for alternative activation of macrophages. Nat Immunol.

[6] Chistiakov DA, Melnichenko AA, Myasoedova VA, Grechko AV, Orekhov AN (2017). Mechanisms of foam cell formation in atherosclerosis. J Mol Med.

[7] Tang Y, Zhou J, Hooi SC, Jiang Y-M, Lu G-D (2018). Fatty acid activation in carcinogenesis and cancer development: essential roles of long-chain acyl-CoA synthetases. Oncol Lett.

[8] Szeto HH (2017). Pharmacologic approaches to improve mitochondrial function in AKI and CKD. J Am Soc Nephrol..

[9] Malandrino MI, Fucho R, Weber M, Calderon-Dominguez M, Mir JF, Valcarcel L (2015). Enhanced fatty acid oxidation in adipocytes and macrophages reduces lipid-induced triglyceride accumulation and inflammation. Am J Physiol Endocrinol Metab.

[10] Gibson MS, Domingues N, Vieira OV (2018). Lipid and non-lipid factors affecting macrophage dysfunction and inflammation in atherosclerosis. Front Physiol.

[11] Testa G, Rossin D, Poli G, Biasi F, Leonarduzzi G (2018). Implication of oxysterols in chronic inflammatory human diseases. Biochimie.

[12] Hayden JM, Brachova L, Higgins K, Obermiller L, Sevanian A, Khandrika S, Reaven P (2002). Induction of monocyte differentiation and foam cell formation in vitro by 7-ketocholesterol. J Lipid Res.

[13] Xue J, Schmidt SV, Sander J, Draffehn A, Krebs W, Quester I (2014). Transcriptome-based network analysis reveals a spectrum model of human macrophage activation. Immunity.

[14] Nahrendorf M, Swirski FK (2016). Abandoning M1/M2 for a network model of macrophage function. Circ Res.

[15] Poli G, Biasi F, Leonarduzzi G (2013). Oxysterols in the pathogenesis of major chronic diseases. Redox Biol.

[16] Sallam T, Jones M, Thomas BJ, Wu X, Gilliland T, Qian K (2018). Transcriptional regulation of macrophage cholesterol efflux and atherogenesis by a long noncoding RNA. Nat Med.

[17] Terasaka N, Wang N, Yvan-Charvet L, Tall AR (2007). High-density lipoprotein protects macrophages from oxidized low-density lipoprotein-induced apoptosis by promoting efflux of 7-ketocholesterol via ABCG1. Proc Natl Acad Sci.

[18] Hong C, Walczak R, Dhamko H, Bradley MN, Marathe C, Boyadjian R (2011). Constitutive activation of LXR in macrophages regulates metabolic and inflammatory gene expression: identification of ARL7 as a direct target. J Lipid Res.

[19] Oh J, Riek AE, Weng S, Petty M, Kim D, Colonna M (2012). Endoplasmic reticulum stress controls M2 macrophage differentiation and foam cell formation. J Biol Chem.

[20] Ménégaut L, Thomas C, Lagrost L, Masson D (2017). Fatty acid metabolism in macrophages: a target in cardio-metabolic diseases. Curr Opin Lipidol.

[21] Nomura M, Liu J, Rovira II, Gonzalez-Hurtado E, Lee J, Wolfgang MJ (2016). Fatty acid oxidation in macrophage polarization. Nat Immunol.

[22] Diskin C, Pålsson-McDermott EM (2018). Metabolic modulation in macrophage effector function. Front Immunol.

[23] Kaseda R, Tsuchida Y, Yang HC, Yancey PG, Zhong J, Tao H (2018). Chronic kidney disease alters lipid trafficking and inflammatory responses in macrophages: Effects of liver X receptor agonism. BMC Nephrol.

[24] Qu Q, Zeng F, Liu X, Wang QJ, Deng F (2016). Fatty acid oxidation and carnitine palmitoyltransferase I: emerging therapeutic targets in cancer. Cell Death Dis.

[25] Bentebibel A, Sebastián D, Herrero L, López-Viñas E, Serra D, Asins G (2006). Novel effect of C75 on carnitine palmitoyltransferase I activity and palmitate oxidation. Biochemistry.

[26] Mera P, Bentebibel A, López-Viñas E, Cordente AG, Gurunathan C, Sebastián D (2009). C75 is converted to C75-CoA in the hypothalamus, where it inhibits carnitine palmitoyltransferase 1 and decreases food intake and body weight. Biochem Pharmacol.

[27] Duewell P, Kono H, Rayner KJ, Sirois CM, Vladimer G, Bauernfeind FG (2010). NLRP3 inflammasomes are required for atherogenesis and activated by cholesterol crystals. Nature.

[28] Cantuti-Castelvetri L, Fitzner D, Bosch-Queralt M, Weil M-T, Su M, Sen P (2018). Defective cholesterol clearance limits remyelination in the aged central nervous system. Science.

[29] Moon J-S, Nakahira K, Chung K-P, DeNicola GM, Koo MJ, Pabón MA (2016). NOX4-dependent fatty acid oxidation promotes NLRP3 inflammasome activation in macrophages. Nat Med.

